# Comprehensive circulating tumor DNA mutation profiling via CAPP-Seq liquid biopsy for cervical cancer

**DOI:** 10.1007/s10147-026-03006-1

**Published:** 2026-03-10

**Authors:** Naoyuki Iwahashi, Tomoko Noguchi, Kazuko Sakai, Tamaki Yahata, Kaho Nishioka, Megumi Fujino, Shinichiro Takeda, Nobuhiko Suzuki, Kazuto Nishio, Kazuhiko Ino

**Affiliations:** 1 Department of Obstetrics and Gynecology, School of Medicine, Wakayama Medical University, 811-1 Kimiidera, Wakayama, 641-8509 Japan; 2 Faculty of Medicine, Department of Genome Biology, Kindai University, Osaka, Japan

**Keywords:** Liquid biopsy, Circulating tumor DNA, Comprehensive genomic profiling, Cervical cancer

## Abstract

**Background:**

Liquid biopsy using circulating tumor DNA (ctDNA) is a minimally invasive approach for detecting tumor-associated genomic alterations. Although ctDNA analysis has been widely explored in solid tumors, its application to cervical cancer remains limited. Cancer personalized profiling by deep sequencing (CAPP-Seq) enables sensitive ctDNA profiling via molecular barcoding and digital error suppression.

**Methods:**

We evaluated the feasibility of ctDNA-based mutation profiling in cervical cancer using the CAPP-Seq platform by analyzing plasma samples from 38 patients.

**Results:**

The cohort included three patients with stage I disease, nine with stage II, 19 with stage III, and seven with stage IV. Somatic gene alterations were detected in 33 of the 38 cases (87%), including squamous cell carcinoma (27/29 [93%]) and adenocarcinoma (6/9 [67%]). Non-synonymous mutations were identified in 23 patients (59%), with *PIK3CA* being the most frequently mutated gene [13/38 (34%)]. Copy number gains of *EGFR*, *MET*, and *ERBB2* were observed in 24%, 11%, and 5% of cases, respectively. The median blood tumor mutational burden was 17.7 mutations/Mb, and 50% of the patients exhibited a hypermutated phenotype. In a subset of four patients who received concurrent chemoradiotherapy, longitudinal changes in ctDNA mutation profiles between pre- and post-treatment samples were associated with treatment response.

**Conclusions:**

This study demonstrates the feasibility of ctDNA-based mutation profiling using CAPP-Seq in cervical cancer, with a high detection rate of tumor-associated genomic alterations across histological subtypes. ctDNA analysis may represent a minimally invasive approach for the molecular characterization and disease monitoring of cervical cancer.

## Introduction

Cervical cancer remains a major global health challenge, with hundreds of thousands of new cases diagnosed each year and significant mortality rates at advanced stages [1, 2]. Despite advances in diagnostic imaging, surgical techniques, and multimodal therapy approaches, a significant proportion of patients present with locally advanced disease or eventually develop recurrence. Early detection of primary, persistent, or recurrent lesions is vital for tailoring interventions, such as surgery, radiotherapy, or chemotherapy. However, the limitations of existing diagnostic modalities underscore the need for more sensitive, minimally invasive, and real-time methods to evaluate disease status. Early detection of residual or recurrent disease is often difficult because imaging may fail to discern small tumor foci or micrometastases, and serum markers may lack adequate specificity or dynamic range for individualized treatment monitoring. Consequently, there is an urgent need for noninvasive biomarkers capable of providing timely, patient-specific insights into disease progression and response to therapy.

Liquid biopsy, which measures tumor-derived components, such as circulating tumor DNA (ctDNA), in the bloodstream, has gained increasing attention as a means of characterizing the molecular landscape of tumors in real time [3]. These components include circulating tumor cells (CTCs), ctDNA, and other tumor-related analytes. Among these, ctDNA has gained particular prominence because it carries specific genetic and epigenetic alterations found in primary and metastatic tumors. As ctDNA mirrors the genetic and epigenetic attributes of both primary and metastatic tumors, it provides real-time molecular information without the drawbacks of repeated tissue biopsies. Although ctDNA-based methods have been extensively studied in various solid tumors, including lung, breast, and colorectal cancers—such as in minimal residual disease detection, therapeutic monitoring, and selection of targeted treatments [4, 5]—few studies have focused on gynecologic malignancies, particularly cervical cancer.

The effective implementation of ctDNA assays in cervical cancer requires highly sensitive methodologies capable of detecting trace amounts of tumor-derived genetic material within abundant non-malignant cell-free DNA [6]. Earlier protocols, such as digital polymerase chain reaction (dPCR), were constrained by their limited capacity for broad genomic analysis. More recent advances, such as cancer personalized profiling by deep sequencing (CAPP-seq), harness molecular barcoding combined with error-suppression algorithms to achieve sensitive and wide-ranging mutation detection [7]. Although this ultrasensitive approach has been employed for various cancers [8–12], its comprehensive utility in cervical cancer has not yet been elucidated. In this study, we used CAPP-seq to comprehensively profile ctDNA-derived genetic changes in cervical cancer. We also investigated whether these molecular alterations correlated with clinicopathological parameters, disease stage, and survival outcomes, thereby exploring the feasibility of using ctDNA to guide personalized therapeutic strategies.

## Patients and methods

### Participants

We retrospectively enrolled 38 women (median age 61 years) newly diagnosed with cervical cancer who underwent primary surgical resection or concurrent chemoradiotherapy (CCRT) (50 Gy radiotherapy combined with six weekly cycles of cisplatin at 40 mg/m^2^) between July 2017 and August 2020 at Wakayama Medical University Hospital, Japan. Staging was based on International Federation of Gynecology and Obstetrics (FIGO) criteria, and histopathological classification followed World Health Organization (WHO) guidelines. For CCRT patients, imaging modalities including computed tomography (CT), magnetic resonance imaging (MRI), and positron emission tomography/computed tomography (PET/CT) were employed for clinical staging. Adjuvant or recurrent chemotherapy included three to six cycles of paclitaxel (175 mg/m^2^) and carboplatin (AUC = 5.0) every 3–4 weeks. Blood specimens were obtained prior to initial therapy, and for CCRT recipients, both pre- and post-treatment blood samples were collected.

Approval was granted by the ethical committees of Wakayama Medical University (authorization number: 2026) and Kindai University Faculty of Medicine (authorization number: 29-066). Written informed consent was obtained from each subject, and the study adhered to the principles of the 2008 revision of the Declaration of Helsinki.

### Circulating tumor DNA extraction

For cell-free DNA (cfDNA) isolation, peripheral blood (8.5 mL) was drawn into cell-free DNA blood collection tubes (Roche Diagnostics, Indianapolis, IN, USA). From each sample, 4 mL of plasma was processed with the AVENIO cfDNA isolation kit (Roche Diagnostics) according to the manufacturer’s protocol, and ctDNA was eluted in 65 µL of elution buffer. Concentration and quality of the extracted ctDNA were verified using the PicoGreen dsDNA assay kit (Life Technologies, Carlsbad, CA, USA) according to the manufacturer’s protocol. DNA mass measured in nanograms was converted to genome equivalents assuming that one haploid human genome corresponds to 3.3 pg of DNA (approximately 303 genome equivalents per ng), as recommended in the manufacturer’s documentation and commonly applied in clinical cfDNA studies. Samples were stored at – 80 °C until use.

### Circulating tumor DNA sequencing

CAPP-Seq was performed on 10–50 ng of ctDNA using the AVENIO ctDNA Surveillance Kit, which targets 197 genes (Roche Diagnostics), as previously described [11, 12]. The targeted 197-gene CAPP-Seq panel used in this study did not include genes associated with clonal hematopoiesis of indeterminate potential (CHIP). Purified libraries were then pooled and sequenced on an Illumina NextSeq 500 system with a 300-cycle high-output kit (Illumina, San Diego, CA, USA). Variant calling utilized the AVENIO ctDNA Analysis Software (Roche Diagnostics), which integrates the CAPP-Seq pipeline [7] and digital error suppression [13]. Variants found at ≥ 1% frequency in the Exome Aggregation Consortium were eliminated, and only non-synonymous single-nucleotide variants (SNVs), insertions/deletions (Indels), copy-number alterations (CNAs), and gene fusions related to the 197 genes were reported. Blood tumor mutational burden (bTMB) was determined as the number of non-synonymous mutations per megabase.

### Tumor DNA extraction

Tumor DNA was isolated from formalin-fixed paraffin-embedded (FFPE) tissues using the AllPrep DNA/RNA FFPE kit (Qiagen, Inc., Valencia, CA, USA) per the manufacturer’s guidelines. Concentration and purity were assessed by a NanoDrop 2000 spectrophotometer (Thermo Scientific, Wilmington, DE, USA) and PicoGreen dsDNA assay kit (Thermo Fisher Scientific, Waltham, MA, USA). DNA aliquots were kept at – 80 °C until subsequent steps.

### Tumor DNA sequencing

NGS libraries were prepared according to the manufacturer’s protocol with the Ion AmpliSeq Library kit version 2.0 (Thermo Fisher Scientific). For DNA sequencing, 10 ng of DNA was subjected to multiplex PCR amplification with the use of an Ion AmpliSeq Cancer Hotspot Panel (CHPv2) primer pool (50 genes, 207 amplicons; Thermo Fisher Scientific). After multiplex PCR, Ion Xpress Barcode Adapters (Thermo Fisher Scientific) were ligated to the PCR products, which were then purified with the use of Agencourt AMPure XP beads (Beckman Coulter, Brea, CA, USA). The purified libraries were pooled and then sequenced with the use of an Ion Torrent S5 instrument and Ion 550 Chip Kit (all from Thermo Fisher Scientific). DNA sequencing data were accessed through the Torrent Suite v.5.12 program (Thermo Fisher Scientific). Reads were aligned against the hg19 human reference genome, and variants were called with the use of Variant Call Format ver. 5.12. Raw variant calls were filtered with depth of coverage of < 19 and were manually checked using the integrative genomics viewer (IGV; Broad Institute, Cambridge, MA, USA). Germline mutations were excluded with the use of the Human Genetic Variation Database (http://www.genome.med.kyoto-u.ac.jp/SnpDB) [14].

### Immunohistochemistry

Immunohistochemical (IHC) analysis was performed in all cases in which copy-number gains were detected by ctDNA analysis. FFPE tissues of cervical cancer blocks were cut into 3-μm-thick sections, after which sections were deparaffinized and rehydrated. Epitopes were then retrieved by means of heat-induced antigen retrieval—boiling the sections in a pressure cooker in citrate buffer (10 mM sodium citrate, 0.05% Tween 20, pH 6.0) for 20 min. IHC was performed on a Ventana Benchmark XT autostainer (Ventana Medical Systems, Tucson, AZ, USA), according to the manufacturer’s protocol. The primary antibodies used were anti-EGFR mouse monoclonal (clone 3C6, Ventana), anti-c-MET rabbit monoclonal (clone SP44, Ventana), and anti-ERBB2 rabbit monoclonal (clone 4B5, Ventana) antibodies. IHC was scored accordingly: 0, no membrane staining in < 10% tumor cells; 1 + , faint/barely perceptible or weak incomplete membrane staining in > 10% of tumor cells; 2 + , weak to moderate complete membrane staining in > 10% of tumor cells or strong complete membrane staining in ≤ 10% of tumor cells; 3 + , strong complete membrane staining in > 10% of tumor cells. Samples that demonstrated IHC levels of 2 + or 3 + for EGFR, MET, and ERBB2 were classified as positive for overexpression. All slides were independently evaluated by experienced pathologists who were blinded to the ctDNA results.

### Statistical analysis

Comparisons among the four groups were performed using the Kruskal–Wallis test, followed by Dunn’s multiple comparison test. Comparisons between two groups were conducted using the Wilcoxon rank-sum test. Overall survival was estimated using the Kaplan–Meier method, and differences between groups were assessed using the log-rank test. Cox proportional hazards models were used to evaluate the association between variables and survival outcomes. All statistical tests were two-sided, and a *P* value < 0.05 was considered statistically significant. All statistical analyses were conducted using Prism software (GraphPad Software, version 10.6.1; San Diego, CA, USA).

## Results

### Clinical characteristics of the study cohort

We retrospectively studied 38 individuals diagnosed with cervical cancer, including 29 with squamous cell carcinoma (SCC) and nine with adenocarcinoma (Adeno). Relevant details regarding tumor stage, treatment regimens, and histopathological features are summarized in Table 1. Specifically, three patients presented with stage I disease, nine with stage II, 19 with stage III, and seven with stage IV. Pretreatment plasma samples were available from all 38 patients who underwent either surgical resection or CCRT comprising 50 Gy of radiotherapy plus six weekly cycles of cisplatin at 40 mg/m^2^. For adjuvant or recurrent chemotherapy, participants received three to six cycles of paclitaxel (175 mg/m^2^) combined with carboplatin (area under the curve 5.0, per Calvert’s formula) every 3–4 weeks.

**Table 1:** Clinicopathological characteristics of patients with cervical cancer

	All	Squamous cell carcinoma	Adenocarcinoma
	(*n* = 38)	(*n* = 29)	(*n* = 9)
Age, years (mean ± SD)	61.2 ± 15.4	66.3 ± 14.5	59.6 ± 15.3
BMI, kg/m^2^ (mean ± SD)	22.5 ± 4.1	22.1 ± 3.3	22.7 ± 4.3
Primary tumor size, mm (mean ± SD)	48.1 ± 16.7	47.8 ± 15.4	49.3 ± 20.8
*FIGO stage*, *n* (%)			
I	3 (7.9)	2 (6.9)	1 (11.1)
II	9 (23.7)	6 (20.7)	3 (33.3)
III	19 (50.0)	17 (58.6)	2 (22.2)
IV	7 (18.4)	4 (13.8)	3 (33.3)
*Primary therapy*, *n* (%)			
CCRT	28 (73.7)	24 (82.8)	4 (44.4)
Surgical resection	10 (26.3)	5 (17.2)	5 (55.5)
Death at the observation time point, *n* (%)	9 (23.7)	6 (20.7)	3 (33.3)
PFS, days [median (range)]	335 (32–2143)	454 (32–2050)	1163 (49–2143)
OS, days [median (range)]	1148 (42–2143)	187 (42–2050)	341 (49–2143)

*BMI* body mass index, *CCRT* concurrent chemoradiotherapy, *PFS* progression-free survival, *OS* overall survival

### Associations of ctDNA-derived metrics with clinicopathological characteristics

**Fig. 1:**
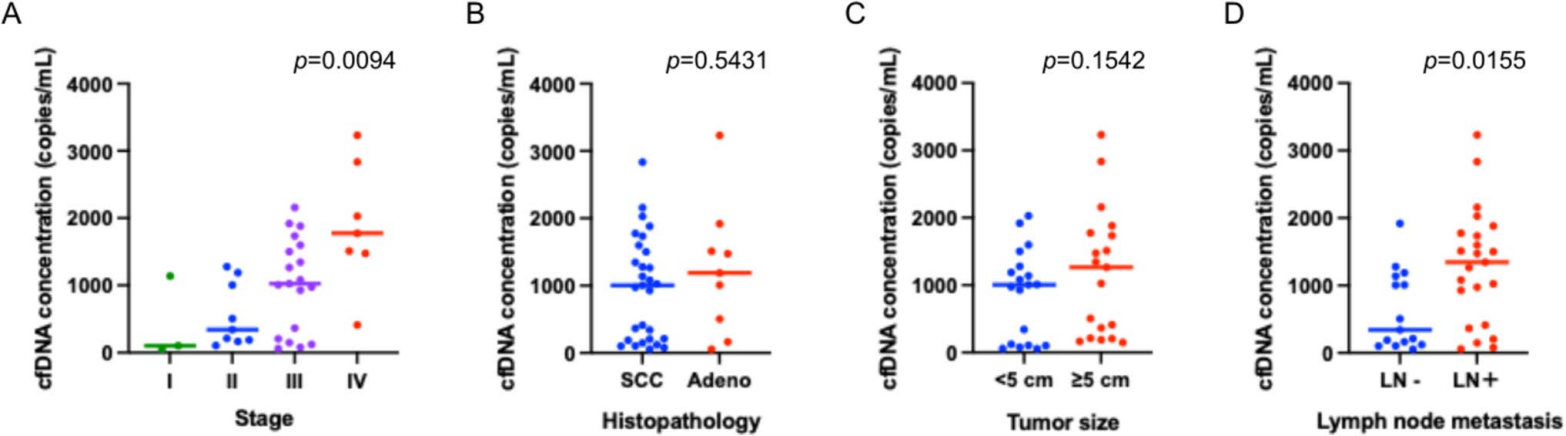
Distribution of pretreatment plasma cell-free DNA (cfDNA) concentration according to clinicopathological characteristics in cervical cancer. **A** cfDNA concentration among disease stages; **B** cfDNA concentration among histologic subtypes; **C** cfDNA concentration among primary tumor size (< 5 cm vs. ≥ 5 cm); **D** cfDNA concentration among lymph node metastasis status. cfDNA concentration is expressed as genome equivalents per milliliter, calculated from PicoGreen-measured DNA mass according to the manufacturer’s protocol. Statistical comparisons were performed using the Kruskal–Wallis test for comparisons among four groups (**A**) and the Wilcoxon rank-sum test for comparisons between two groups (**B**–**D**)

We evaluated the cfDNA concentration, bTMB, and variant allele frequency (VAF) in the pretreatment plasma to assess their association with disease stage, histologic subtype, primary tumor size, and lymph node metastasis (Fig. 1). Comparisons according to the primary tumor size were performed by stratifying the patients into two groups based on a median tumor size of 5 cm. The median cfDNA level was 1016.9 copies/mL (range 55.8–3232.5), and cfDNA concentrations differed significantly among the four stages overall (Kruskal–Wallis test, *p* = 0.0094) (Fig. 1A). Pairwise comparisons using Dunn’s multiple comparison test demonstrated significantly higher cfDNA concentrations in stage IV disease compared with stage I (*p* = 0.033) and stage II (*p* = 0.021). No significant correlation was observed between cfDNA concentration and histological subtype or primary tumor size, whereas cfDNA levels were significantly elevated in patients with lymph node metastases (Fig. 1B–D).

**Fig. 2:**
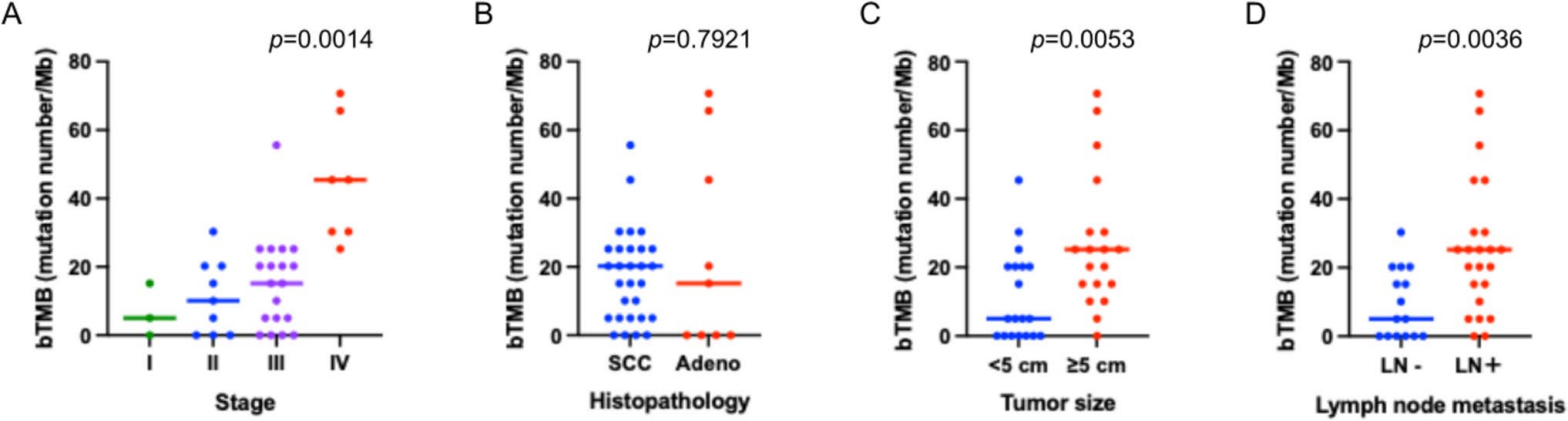
Distribution of pretreatment plasma blood tumor mutational burden (bTMB) according to clinicopathological characteristics in cervical cancer. **A** bTMB concentration among disease stages; **B** bTMB concentration among histologic subtypes; **C** bTMB concentration among primary tumor size (< 5 cm vs. ≥ 5 cm); **D** bTMB concentration among lymph node metastasis status. bTMB is expressed as the number of somatic mutations per megabase (mutations/Mb), derived from ctDNA sequencing data obtained by Cancer Personalized Profiling by Deep Sequencing (CAPP-Seq) analysis. Statistical comparisons were performed using the Kruskal–Wallis test for comparisons among four groups (**A**) and the Wilcoxon rank-sum test for comparisons between two groups (**B**–**D**)

Next, we calculated the bTMB, expressed as the number of non-synonymous mutations per megabase (Fig. 2). The median bTMB was 17.7 mutations/Mb (ranging from 0.0 to 70.7), and 19 of 38 patients (50%) exhibited a hypermutated phenotype (bTMB > 16 mutations/Mb). bTMB levels differed significantly among the four stages overall (Kruskal–Wallis test, *p* = 0.0014) (Fig. 2A). Pairwise comparisons using Dunn’s multiple comparison test demonstrated significantly higher bTMB levels in stage IV disease compared with stage I (*p* = 0.016), II (*p* = 0.004), and III (*p* = 0.007). No significant correlation was observed between bTMB and histological subtype, whereas bTMB was significantly elevated in patients with larger primary tumors and those with lymph node metastasis (Fig. 2B–D).

**Fig. 3:**
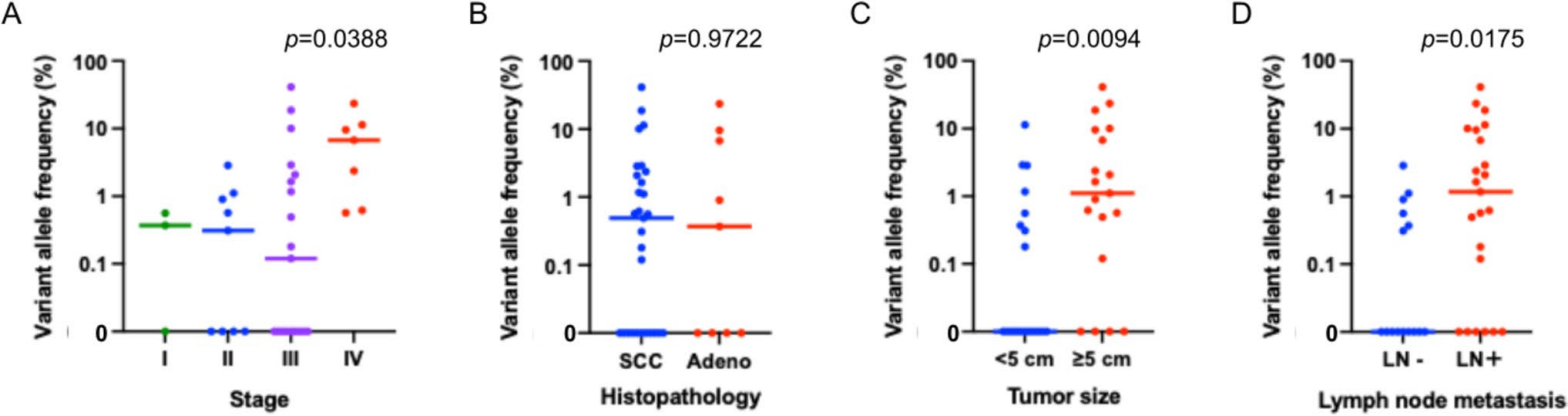
Distribution of pretreatment plasma maximum pathogenic variant allele frequency (VAF) according to clinicopathological characteristics in cervical cancer. **A** Maximum pathogenic VAF among disease stages; **B** maximum pathogenic VAF among histologic subtypes; **C** maximum pathogenic VAF among primary tumor size (< 5 cm vs. ≥ 5 cm); **D** maximum pathogenic VAF among lymph node metastasis status. Maximum pathogenic VAF is defined as the maximum VAF of non-synonymous pathogenic mutations detected in each plasma sample. Statistical comparisons were performed using the Kruskal–Wallis test for comparisons among four groups (**A**) and the Wilcoxon rank-sum test for comparisons between two groups (**B**–**D**)

We examined the detection rates of pathogenic mutations and VAF. For the CAPP-Seq ctDNA analysis, positivity was defined as the presence of non-synonymous mutations with VAF ≥ 0.10. The detection rates of pathogenic mutations according to disease stage were 67% (2/3) for stage I, 56% (5/9) for stage II, 56% (10/19) for stage III, and 100% (7/7) for stage IV. No significant difference in the detection rate of pathogenic mutations was observed according to histological subtype, whereas the detection rate was significantly higher in patients with larger primary tumors (*p* = 0.020) and those with lymph node metastasis (*p* = 0.037). We then focused on the level of the maximum pathogenic VAF among all detected pathogenic mutations in each sample (Fig. 3). Maximum pathogenic VAF differed significantly among the groups overall (Kruskal–Wallis test, *P* = 0.0388) (Fig. 3A), but pairwise comparisons using Dunn’s multiple comparison test did not show statistically significant differences between individual groups. No significant correlation was observed between the maximum pathogenic VAF and histological subtype, whereas the maximum pathogenic VAF was significantly elevated in patients with larger primary tumors and those with lymph node metastasis (Fig. 3B–D).

### Profiling of genetic somatic mutation in cervical cancer via CAPP-Seq

**Fig. 4:**
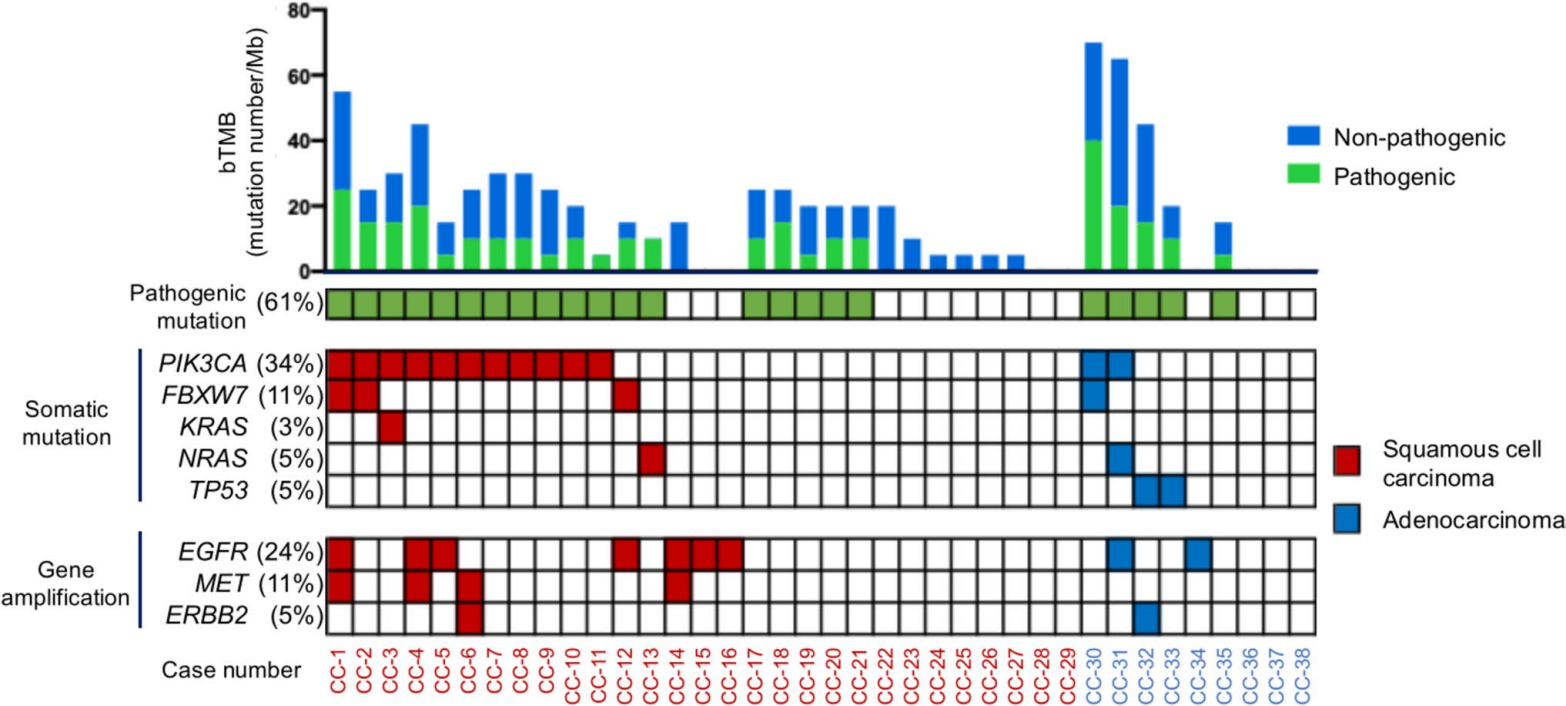
Comprehensive profiling of genetic alterations in 38 patients with cervical cancer using liquid biopsy-based cancer personalized profiling by deep sequencing (CAPP-Seq). The figure summarizes pretreatment plasma-based genomic profiling, including blood tumor mutational burden (bTMB), the presence or absence of pathogenic mutations, and major somatic mutations and copy-number alterations detected by ctDNA analysis. Each column represents an individual patient, and genetic alterations are displayed according to gene and alteration type

Next, we conducted comprehensive genomic profiling of ctDNAs in pretreatment plasma samples using a 197-gene CAPP-seq panel. A complete array of identified alterations, covering pathogenic and non-pathogenic mutations and gene amplification, is shown in Fig. 4. Overall, non-synonymous somatic changes were detected in 33 of 38 patients (87%) [SCC 27/29 (93%), Adeno 6/9 (67%)]. Among these, 23 (61%) carried ≥ 1 pathogenic mutation, most frequently in *PIK3CA* [13/38 (34%)], *FBXW7* [4/38 (11%)], *TP53* [2/38 (5%)], *NRAS* [2/38 (5%)], and *KRAS* [1/38 (3%)], which was concordant with TCGA database [15]. Two patients harbored double *PIK3CA* mutations, suggesting intratumoral heterogeneity.

We also detected somatic copy-number amplification in three genes, *EGFR*, *MET*, and *ERBB2*, in plasma ctDNA using CAPP-Seq. *EGFR* copy-number amplifications were observed in nine patients (24%), *MET* copy-number amplifications were observed in four patients (11%), and *ERBB2* copy-number amplifications were observed in two patients (5%).

**Fig. 5:**
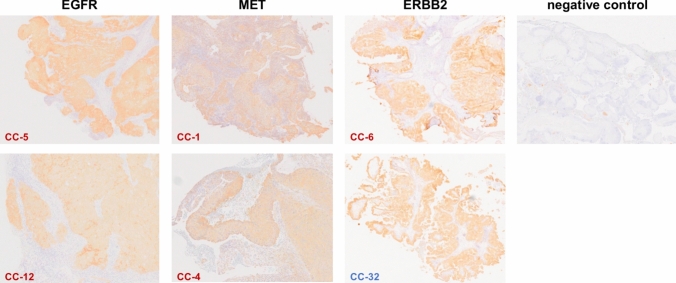
Immunohistochemical staining for EGFR, MET, and ERBB2 in amplification-positive and negative control cases. Representative amplification-positive cases (two cases per gene) and negative control cases are shown. All cases with gene amplification detected by ctDNA analysis were evaluated by immunohistochemistry using corresponding FFPE tumor tissue sections. Protein expression positivity was defined based on membranous staining intensity-based scoring criteria

To evaluate the biological relevance of gene amplification detected by ctDNA analysis, IHC analyses were performed for all amplification-positive cases using the corresponding FFPE tumor tissue blocks. In all cases with detected gene amplification, IHC demonstrated increased protein expression of EGFR, MET, and ERBB2, which was consistent with the ctDNA-based amplification findings (Fig. 5). Although copy-number gains of *EGFR*, *MET*, or *ERBB2* were detected using ctDNA analysis, tissue-based confirmation by in situ hybridization methods such as chromogenic or fluorescence in situ hybridization (CISH/FISH) was not performed in the present study. Given the technical challenges associated with calling gene amplification from ctDNA, additional validation at the genomic level in tumor tissue would improve the robustness of these findings and should be addressed in future studies.

### Concordance of *PIK3CA* mutations between ctDNA and tissue DNA

To validate the concordance of genomic alterations between ctDNA and tissue DNA, we analyzed the tissue DNA of 25 tissue-available cervical cancer patients using Ion AmpliSeq Cancer Hotspot Panel v2 (CHPv2) (50 genes, 207 amplicons; Life Technologies). We focused on *PIK3CA* mutations that were found most in patients with cervical cancer using CAPP-Seq. In this cohort, *PIK3CA* mutations in ctDNA and tissue DNA were detected in 10/25 patients (40%) and 12/25 patients (48%), respectively (Table 2). Synchronous *PIK3CA* mutations were identified in both tumor tissue DNA and ctDNA in 10/12 patients (83%), demonstrating high concordance between plasma- and tissue-based analyses. This concordance supports the validity of the ctDNA assay for detecting tumor-derived genomic alterations in cervical cancer. Interestingly, in two *PIK3CA* double-mutated cases, the second *PIK3CA* mutation was detected only in ctDNA, suggesting that liquid biopsy may capture additional mutations reflecting tumor heterogeneity.

**Table 2:** Concordance of *PIK3CA* mutations between ctDNA and tissue DNA

Case	*PIK3CA* ctDNA	*PIK3CA* tissue DNA	Concordance
CC-1	p.E542K	p.E542K	Match
CC-2	p.E545K	p.E545K	Match
CC-4	p.E545K, p.H1047R*	p.E545K	–
CC-5	p.E542K	p.E542K	Match
CC-6	p.E542K	p.E542K	Match
CC-7	p.E545K, p.E542K*	p.E545K	–
CC-9	p.E545K	p.E545K	Match
CC-11	p.E545G	p.E545G	Match
CC-12	–	–	Match
CC-13	–	–	Match
CC-14	–	–	Match
CC-15	–	–	Match
CC-17	–	–	Match
CC-18	–	p.E542K**	–
CC-19	–	–	Match
CC-24	–	–	Match
CC-25	–	–	Match
CC-28	–	p.E545K**	–
CC-30	p.H1047R	p.H1047R	Match
CC-31	p.E545K	p.E545K	Match
CC-32	–	–	Match
CC-33	–	–	Match
CC-35	–	–	Match
CC-36	–	–	Match
CC-37	–	–	Match

*only in ctDNA, **only in tissue DNA

### Clinical impact of ctDNA-based genetic findings

**Fig. 6:**
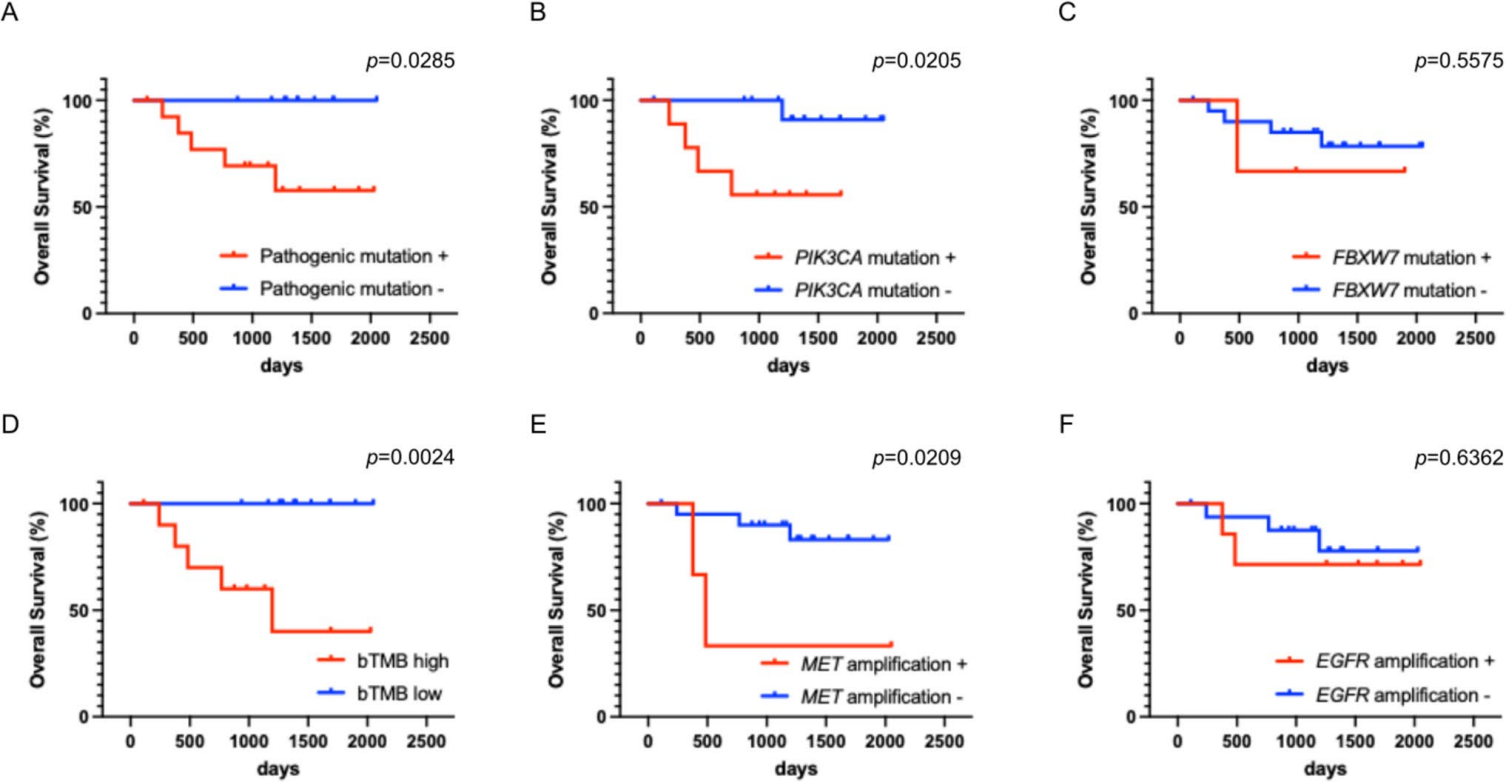
Kaplan–Meier analyses of overall survival (OS) in patients with locally advanced squamous cell carcinoma of the cervix treated with concurrent chemoradiotherapy (CCRT) (*n* = 24). **A** OS stratified by pathogenic mutation status; **B** OS stratified by *PIK3CA* mutation status; **C** OS stratified by *FBXW7* mutation status; **D** OS stratified by blood tumor mutational burden (bTMB) status; **E** OS stratified by *MET* amplification status; **F** OS stratified by *EGFR* amplification status. Survival curves were estimated using the Kaplan–Meier method and compared using the log-rank test

To determine whether the ctDNA-based genetic profile was associated with the clinical outcome of the 38 patients included in the study, a subgroup prognostic analysis was performed focusing on 24 patients with locally advanced SCC treated with CCRT (Table 3). We found that patients harboring at least one pathogenic mutation had significantly shorter overall survival than those without such mutations (Fig. 6A). Among these pathogenic alterations, the presence of *PIK3CA* mutations was significantly associated with clinical outcome, whereas no significant association was observed for *FBXW7* mutations (Fig. 6B, C). Additionally, bTMB was significantly associated with overall survival (Fig. 6D). With respect to gene-specific copy-number alterations, *MET* amplification was significantly associated with poor outcomes after CCRT, whereas no such association was observed for *EGFR* amplification (Fig. 6E, F). Taken together, these results suggest that pathogenic mutation status, bTMB, *PIK3CA* mutation status, and *MET* amplification may have potential prognostic relevance in cervical cancer. However, none of these variables remained statistically significant in the multivariate model, indicating that they were not independent prognostic factors after adjustment.

**Table 3:** Clinicopathological characteristics of patients with squamous cell carcinoma treated with concurrent chemoradiotherapy

Squamous cell carcinoma (*n* = 24)	
Age, years (mean ± SD)	58.2 ± 14.7
BMI, kg/m2 (mean ± SD)	22.7 ± 4.3
Primary tumor size, mm (mean ± SD)	46.5 ± 15.7
*FIGO stage*, n (%)	
I	2 (8.3)
II	6 (25.0)
III	14 (58.3)
IV	2 (8.3)
Death at the observation time point, *n* (%)	5 (20.8)
PFS, days [median (range)]	762 (32–2050)
OS, days [median (range)]	1265 (111–2050)

*BMI* body mass index, *CCRT* concurrent chemoradiotherapy, *PFS* progression-free survival, *OS* overall survival

### Longitudinal monitoring of ctDNA

**Fig. 7:**
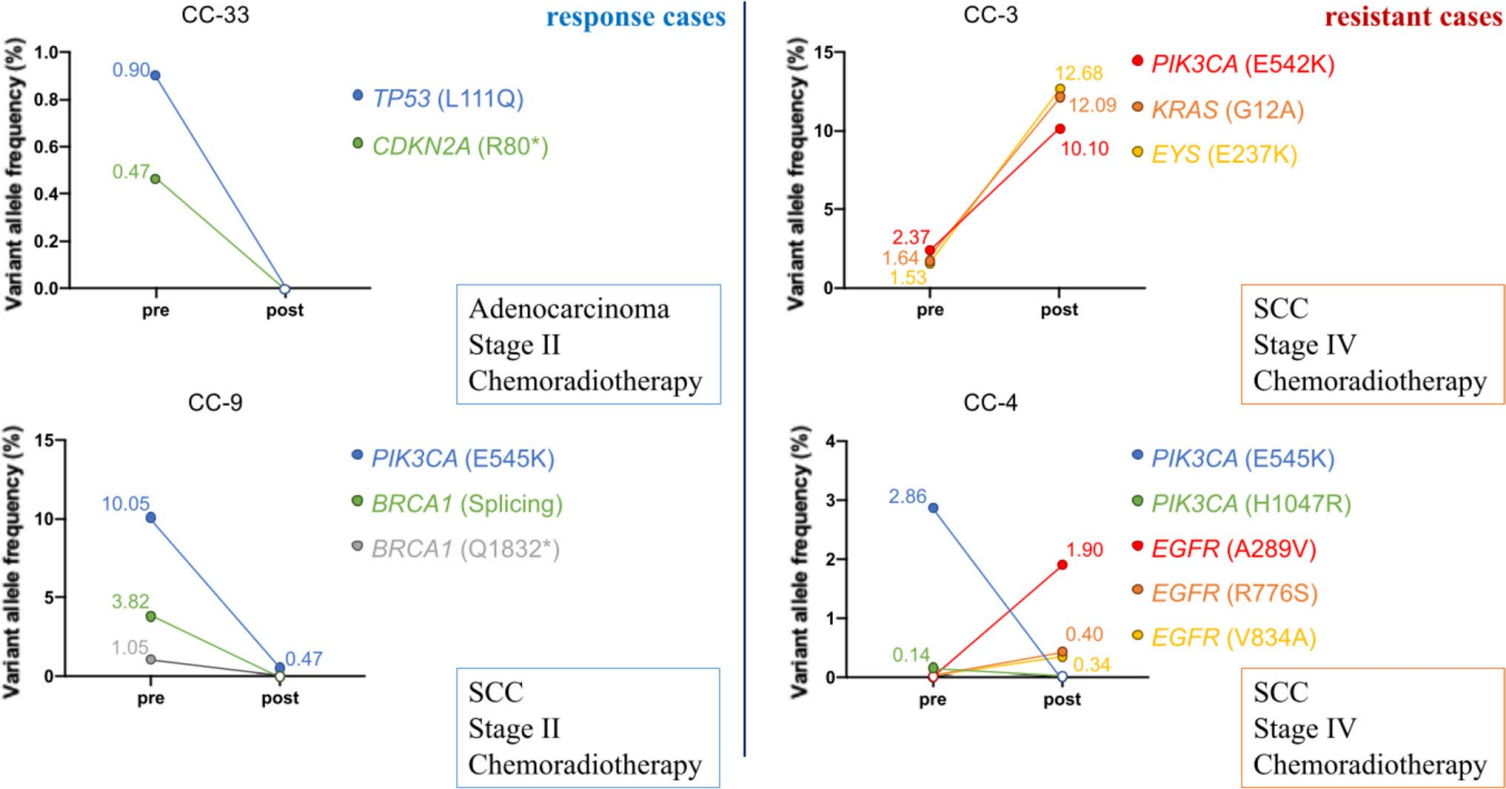
Longitudinal monitoring of circulating tumor DNA (ctDNA) during concurrent chemoradiotherapy (CCRT) in cervical cancer. Representative longitudinal changes in ctDNA mutation profiles before and after CCRT are shown for four patients with paired plasma samples. Two patients with favorable treatment response (CC-33 and CC-9) and two patients with treatment resistance (CC-3 and CC-4) are presented. For each case, non-synonymous pathogenic somatic mutations detected by CAPP-Seq are displayed, with variant allele fractions (VAFs) compared between pre- and post-CCRT samples

To evaluate dynamic changes during CCRT, we compared pre- and post-CCRT ctDNA in four illustrative cases (Fig. 7) with cervical cancer: two CCRT-response cases (CC-33, CC-9) and two CCRT-resistance cases (CC-3, CC-4) were analyzed. CC-33 was diagnosed as stage II Adeno and treated with CCRT. *TP53* (p.L111Q) and *CDKN2A* (p.R80*) mutations in the pre-CCRT ctDNA were not detectable post-CCRT. CC-9 was diagnosed as stage II SCC and treated with CCRT. The allele fraction of *PIK3CA* (p.E545K) mutation decreased in post-CCRT ctDNA compared with pre-CCRT ctDNA, and the *BRCA1* splicing variant and p.Q1832* mutation in pre-CCRT ctDNA were not detectable post-CCRT. CC-3 was diagnosed as stage IV SCC and treated with CCRT. The allele fractions of *PIK3CA* (p.E542K), *KRAS* (G12A), and *EYS* (E237K) mutations were higher in post-CCRT ctDNA than in pre-CCRT ctDNA. CC-4 was diagnosed as stage IV SCC and treated with CCRT. Notably, although *PIK3CA* (p.E545K, p.H1047R) mutations became undetectable, *EGFR* (p.A289V, p.R776S, p.V834A) mutations arose de novo, highlighting the need for longitudinal ctDNA surveillance to capture clonal shifts.

## Discussion

In this study, we aimed to establish a proof-of-concept that a sensitive ctDNA assay, CAPP-Seq, has the potential to complement existing diagnostic and therapeutic monitoring strategies for cervical cancer. In our previous study, we showed, for the first time, the feasibility of ctDNA analysis using CAPP-Seq in gynecological cancers [11], including larger ovarian cancer cohorts [12]; thus, we extended its application to cervical cancer. Our approach highlights tumor-derived genetic alterations with potential clinical relevance, which might be useful not only as a noninvasive tumor genotyping tool but also as a predictive biomarker of prognosis in clinical situations for cervical cancer.

Although recent advancements in liquid biopsy techniques have significantly enhanced our ability to detect and characterize molecular alterations in cancer treatment strategies, available data on ctDNA in cervical cancer are limited. Interestingly, we demonstrated an association between pathogenic somatic mutations and clinical outcomes, which has been previously reported for other solid cancers [16, 17]. In the present study, patients harboring one or more pathogenic somatic mutations, as defined in the COSMIC database, exhibited significantly poorer overall survival compared with those without pathogenic mutations. In addition, *PIK3CA* mutation status, *MET* amplification, and bTMB were each suggested to contribute to prognosis, indicating that ctDNA-based genomic profiling using CAPP-Seq may have potential value in predicting disease progression in cervical cancer. Although several biomarkers demonstrated significant associations with survival in univariate analyses, none were statistically significant in the multivariate model, suggesting that these factors are not independent prognostic indicators when considered simultaneously. This finding may reflect overlapping biological information captured by these biomarkers, as well as the limited number of events in the present cohort, which may have reduced the statistical power of the multivariable analyses. Therefore, larger prospective studies with systematic sampling, increased event numbers, and external validation are necessary to clarify the independent prognostic contributions of ctDNA-derived biomarkers and determine their potential clinical utility in cervical cancer.

To focus on *PIK3CA*, the most mutated gene in cervical cancer, Chung et al. investigated the prevalence of two *PIK3CA* mutations (p.E542K and p.E545K) in 117 patients with cervical cancer using droplet digital PCR for ctDNA [18]. In this study, either p.E542K or p.E545K *PIK3CA* mutations were detected in plasma ctDNA from 22.2% of patients, and the detection of *PIK3CA* mutations in plasma was associated with reduced disease-free survival and overall survival. Subsequently, Tian et al. used next-generation sequencing (NGS) to detect gene alterations in ctDNA from 57 patients with cervical cancer [19]. Although they used a comprehensive gene panel, the detection rate of *PIK3CA* mutations was only 7%, which may be a limitation of the normal NGS method for analyzing ctDNA. In our study, one of the most notable outcomes was the high incidence of *PIK3CA* mutations (34%), including double mutations in two cases, owing to the use of CAPP-Seq, an ultrasensitive NGS method for detecting gene alterations in ctDNA. To validate the concordance of genomic alterations between tissue DNA and ctDNA, we analyzed tissue DNA of 25 patients focusing on *PIK3CA* mutations. We found synchronous *PIK3CA* mutations in both tissue DNA and ctDNA in nearly all cases, which suggests that the mutational analysis of ctDNA in our study mostly reflects the tumor mutational landscape. The two cases with double *PIK3CA* mutations may indicate the coverage of tumor heterogeneity by ctDNA liquid biopsy.

Owing to the comprehensive genetic analysis by CAPP-Seq, we obtained other important gene alterations that could be used as biomarkers for molecular-targeted therapy. We examined bTMB, the number of missense mutations per megabase, which might be a surrogate for the overall neoantigen load arising as a result of tumor-specific mutations [20, 21], and attempted to show the correlation between bTMB and clinical outcomes in patients with cervical cancer. Gandara et al. demonstrated that bTMB may be associated with the clinical benefits of immune checkpoint inhibitor therapy in patients with lung cancer [22]. There have been many clinical studies on bTMB, especially in lung cancer [23, 24], but not many in the gynecological field [12, 25]. In our current analysis of bTMB among clinical stages and different histological subtypes, we detected some populations of highly mutated tumors in each group, and the bTMB-high group had a better prognosis than the bTMB-low group among patients with CCRT-treated cervical cancer. Thus, bTMB might serve as a noninvasive alternative biomarker for clinical benefit in patients with cervical cancer, including the estimation of the effect of immune checkpoint inhibitors in advanced stages. In addition to bTMB, we also identified copy-number gains in *EGFR*, *MET*, and *ERBB2* using CAPP-Seq, which is consistent with previously reported protein expression patterns [26]. These copy-number gains could be targets for novel molecular treatments [27–29], thus ctDNA detection of *EGFR*, *MET*, and *ERBB2* amplification is a key step in stratifying patients for biomarker-driven treatment. Taken together, ctDNA-CAPP-Seq has the potential to provide a new strategy for selecting multiple types of molecular treatments for patients with cervical cancer.

Among the four patients undergoing CCRT for whom paired pre- and post-treatment ctDNA samples were available, dynamic changes in gene mutation patterns detected by liquid biopsy were associated with treatment response. Specifically, decreases or disappearance of VAFs of pathogenic mutations were observed in CCRT-responsive cases, whereas increases in VAFs and the emergence of additional pathogenic mutations were noted in CCRT-resistant cases. These findings are consistent with previous reports showing that a marked reduction or clearance of ctDNA after therapy is associated with favorable outcomes, whereas persistently detectable or newly emerging ctDNA is correlated with disease progression or relapse [12, 19]. Notably, in the CCRT-resistant CC-4, the VAFs of two pathogenic *PIK3CA* mutations decreased following treatment, whereas three pathogenic *EGFR* mutations emerged after CCRT. This pattern suggests clonal selection and expansion of resistant subclones under therapeutic pressure, reflecting tumor evolutionary dynamics during treatment. Although these observations support the potential utility of CAPP-Seq-based ctDNA analysis for monitoring treatment responses and tumor evolution in cervical cancer, these findings should be interpreted with caution because of the limited number of patients available for longitudinal analysis. As paired pre- and post-CCRT ctDNA samples were available for only four patients, these results are exploratory and hypothesis generating. Larger prospective studies with systematic longitudinal sampling are required to validate the role of ctDNA analysis in monitoring treatment and capturing tumor evolutionary changes during CCRT.

This study has several limitations. First, tumor tissue DNA was not analyzed using the same CAPP-Seq gene panel, precluding direct evaluation of concordance and sensitivity between tissue- and plasma-based mutation detection. Further experiments using tumor DNA are needed to explore the usability and sensitivity of ctDNA in detecting tumor mutations. Second, this was a retrospective analysis conducted on a relatively small cohort, which may limit the generalizability and statistical power of our findings. Further studies should be conducted with larger numbers of patients to clarify the clinical usefulness of CAPP-Seq-based ctDNA profiling. In addition, matched leukocyte DNA was not available for germline or CHIP filtering. Although the 197-gene CAPP-Seq panel used in this study did not include genes most associated with CHIP, the absence of leukocyte controls prevented the complete exclusion of rare CHIP-related or germline variants. Future studies incorporating paired leukocyte sequencing are important to further improve the accuracy and reliability of ctDNA-based variant calling.

Collectively, our findings suggest that ctDNA liquid biopsy using CAPP-Seq may represent a feasible and minimally invasive approach for molecular profiling of patients with cervical cancer. Although the clinical applicability of this strategy requires further validation, ctDNA-based genomic analyses may provide complementary insights into tumor biology and disease dynamics. As our knowledge of the molecular heterogeneity of cervical cancer continues to evolve, ctDNA-based biomarker research may contribute to the refinement of risk stratification and therapeutic assessment, supporting future efforts toward more individualized disease management.

## Data Availability

The dataset used and/or analyzed during the current study is available from the corresponding author on reasonable request.
